# Melatonin: A Small Molecule but Important for Salt Stress Tolerance in Plants

**DOI:** 10.3390/ijms20030709

**Published:** 2019-02-07

**Authors:** Haoshuang Zhan, Xiaojun Nie, Ting Zhang, Shuang Li, Xiaoyu Wang, Xianghong Du, Wei Tong, Weining Song

**Affiliations:** 1 State Key Laboratory of Crop Stress Biology in Arid Areas, College of Agronomy and Yangling Branch of China Wheat Improvement Center, Northwest A&F University, Yangling 712100, China; zhanhaoshuang@nwsuaf.edu.cn (H.Z.); small@nwsuaf.edu.cn (X.N.); zhangting@nwsuaf.edu.cn (T.Z.); Lishuang@nwsuaf.edu.cn (S.L.); xiaoyuw@nwsuaf.edu.cn (X.W.); xianghongdu@nwsuaf.edu.cn (X.D.); 2 ICARDA-NWSUAF Joint Research Center for Agriculture Research in Arid Areas, Yangling 712100, China

**Keywords:** antioxidant systems, ion homeostasis, melatonin, salt stress, signal pathway

## Abstract

Salt stress is one of the most serious limiting factors in worldwide agricultural production, resulting in huge annual yield loss. Since 1995, melatonin (*N*-acetyl-5-methoxytryptamine)—an ancient multi-functional molecule in eukaryotes and prokaryotes—has been extensively validated as a regulator of plant growth and development, as well as various stress responses, especially its crucial role in plant salt tolerance. Salt stress and exogenous melatonin lead to an increase in endogenous melatonin levels, partly via the phyto-melatonin receptor CAND2/PMTR1. Melatonin plays important roles, as a free radical scavenger and antioxidant, in the improvement of antioxidant systems under salt stress. These functions improve photosynthesis, ion homeostasis, and activate a series of downstream signals, such as hormones, nitric oxide (NO) and polyamine metabolism. Melatonin also regulates gene expression responses to salt stress. In this study, we review recent literature and summarize the regulatory roles and signaling networks involving melatonin in response to salt stress in plants. We also discuss genes and gene families involved in the melatonin-mediated salt stress tolerance.

## 1. Introduction

Salinity represents an environmental stress factor affecting plant growth and development, and a destructive threat to global agricultural production [1], which damages more than 400 million hectares of land—over 6% of the world’s total land area. Of the irrigated farmland areas, currently 19.5% are salt-affected, with increasing numbers facing the threat of salinization (http://www.plantstress.com/Articles/index.asp). The effects of salt stress on plants mainly include osmotic stress, specific ion toxicity, nutritional imbalance, and reactive oxygen species [2]. Osmotic stress is a rapid process caused by salt concentrations around the roots, which is induced at the initial stage of salt stress [1,2,3]. Na^+^ accumulation at a later stage causes nutrient imbalance, leading to specific ion toxicity [4]. Plants’ exposure to salt stress induces overproduction of reactive oxygen species (ROS), which results in membrane injury [5,6].

Melatonin is a multi-regulatory molecule likely to be present in most plants and animals [7]. It was first identified in 1958, in the bovine pineal gland [8], and is a well-known animal hormone regulating various biological processes, such as the circadian rhythm [9,10], antioxidant activity [11], immunological enhancement [12], seasonal reproduction [13], emotional status, and physical conditions [14]. In 1995, melatonin was discovered in vascular plants [15,16], which initiated this field of study. Melatonin was found to have many physiological functions similar to indole-3-acetic acid (IAA), such as regulating plant photoperiod and protecting chlorophyll [17]. More importantly, it acts as a powerful antioxidant, thus protecting plants from various biotic/abiotic stresses [18,19].

In recent years, more functions of melatonin have been identified in higher plants, mainly its roles as a stress responses regulator. In this review, we systematically discuss the functional and potential regulatory mechanisms of melatonin in response to salt stress. We also focus on the putative genes involved in the melatonin-induced salt stress resistance. Furthermore, we summarized plant melatonin receptors, thus outlining the current situation and further directions for promoting the study of plant salt stress tolerance.

## 2. Function and Mechanism of Melatonin Effects on Plant Salt Tolerance

Extensive studies have revealed the crucial and indispensable roles that melatonin plays in increasing salt tolerance in diverse plant species (Table 1). These functions regulate antioxidant systems to protect plants from the salt stress-induced water deficits and physiological damages, improve photosynthetic efficiency and ion homeostasis, and behave as an activator mediating NO signaling and the polyamine metabolism pathway [7,17,33].

### 2.1. Melatonin Activates Antioxidant Systems in Response to Salt Stress

Salinity induces reactive oxygen species (ROS) production, including superoxide anion (O_2_^−^), hydrogen peroxide (H_2_O_2_), hydroxyl radical (OH^−^), and singlet oxygen (^1^O_2_) [47]. Excess ROS usually leads to cell damage and oxidative stress [22]; it also acts as signaling molecules fundamentally involved in mediating salt tolerance [48]. Plants have developed two antioxidant systems to alleviate ROS-triggered damages: the enzymatic and non-enzymatic systems [49]. In response to salt stress, plants have evolved a complex antioxidant enzyme system, including superoxide dismutase (SOD), guaiacol peroxidase (POD), catalase (CAT), glutathione peroxidases (GPX), glutathione S-transferase (GST), dehydroascorbate reductase (DHAR), glutathione reductase (GR), and ascorbate peroxidase (APX) [17]. The non-enzymatic system, including ascorbic acid (AsA), α-tocopherols, glutathione (GSH), carotenoids, and phenolic compounds, is also essential for ROS elimination [50].

Exogenous melatonin treatment significantly reduced salinity-induced ROS. Following 12 days of salt stress, H_2_O_2_ concentration increased by 37.5%, while melatonin pre-treatment of cucumber maintained a low H_2_O_2_ concentration throughout the experiment [17]. Similar results were also observed in salt-stressed rapeseed seedlings, and the application of exogenous melatonin decreased H_2_O_2_ content by 11.2% [36]. Liang et al. [38] discovered inhibitory effects of melatonin resulting in an increased rate of H_2_O_2_ production in rice seedlings under salt stress, showing that melatonin works in a concentration-dependent manner. Melatonin scavenges ROS, mainly triggered by salt stress, via three pathways. Melatonin acts as a broad-spectrum antioxidant that interacts with ROS and directly scavenges it [51]. The primary function of melatonin is to act as a free radical scavenger and an antioxidant. Through the free radical scavenging cascade, a single melatonin molecule can scavenge up to 10 reactive oxygen species (ROS)/reactive nitrogen species (RNS), which differs from other conventional antioxidants [51]. Exogenous melatonin decreases H_2_O_2_ and O_2_^−^ concentrations by activating antioxidant enzymes. This function has been confirmed in many plant species, such as rapeseed, radish, cucumber, rice, maize, bermudagrass, soybean, watermelon, kiwifruit, and *Malus hupehensis* [36]. In cucumber, the activity of major protective antioxidant enzymes—including SOD, CAT, POD, and APX—in melatonin pre-treated plants was significantly higher than control plants [17]. Under salt stress, exogenous melatonin application also significantly increased the activities of APX, CAT, SOD, POD, GR, and GPX in melatonin-treated seedlings compared to their non-treated counterparts [31,33]. Moreover, melatonin interacts with ROS by improving concentrations of antioxidants (AsA-GSH) [17]. In cucumber, AsA and GSH concentrations in melatonin pre-treated plants were 1.7- and 1.3-fold higher, respectively, compared to control plants [17]. Other studies have reported a marked melatonin-dependent induction of AsA and GSH in maize seedlings under salt stress [31]. These findings suggest that exogenous melatonin could activate enzymatic and non-enzymatic antioxidants to scavenge salt stress-induced ROS, thus improving salt stress tolerance in plants.

### 2.2. Melatonin Improves Plant Photosynthesis under Salt Stress

Photosynthesis, an important physio-chemical process responsible for energy production in higher plants, can be indirectly affected by salt stress [46,52]. For many plant species suffering salt stress, decline in productivity is often associated with lower photosynthesis levels [52]. There are two possible reasons for the salt-induced photosynthesis decline: stomatal closure and affected photosynthetic apparatus [52]. Salt stress can cause stomatal closure, and stomatal conductance (Gs) is one of the parameters for evaluating photosynthesis [52]. The parameters of chlorophyll fluorescence include maximum photochemical efficiency of PSII (Fv/Fm), photochemical quenching (qP), non-photochemical quenching [Y(NPQ)], and actual photochemical efficiency of PSII [Y(II), etc. [46].

In addition to its broad-spectrum antioxidant effects, melatonin participates in the regulation of plant photosynthesis under salt stress. Pretreatment with various concentrations (50–500 μM) of melatonin clearly improved salt tolerance in watermelons, where the leaf net photosynthetic rate (Pn), Gs, chlorophyll content, Y(II) and qP were significantly decreased under salt stress. However, this decrease was alleviated by melatonin pretreatment. Melatonin can also protect watermelon photosynthesis by alleviating stomatal limitation [46]. Similar results were observed in salt-stressed cucumber seedlings, where the photosynthetic capacity of cucumber was significantly improved by exogenous melatonin at 50–150 μM concentrations. Photosynthesis improvement is manifested by increased P_N_, maximum quantum efficiency of PSII, and total chlorophyll content [17]. In radish seedling, chlorophyll a, chlorophyll b and total chlorophyll contents increased upon melatonin treatment under salt stress, and the 100 μM dose was the best [34]. Melatonin also enhanced rice seedlings’ salt tolerance by decreasing chlorophyll’s degradation rate [38]. Even though the chlorophyll content in melatonin-treated maize seedlings did not change, an obvious increase in Pn was observed under salt stress [33]. Exogenous melatonin’s protective roles in photosynthesis were also observed in soybean, apple, and tomato [21,40,44]. Overall, exogenous melatonin improves photosynthesis by effectively alleviating chlorophyll degradation and stomatal closure caused by salt stress, therefore enhancing salt stress tolerance.

### 2.3. Melatonin Promotes Ion Homeostasis under Salt Stress

Ion homeostasis refers to the ability of living organisms to maintain stable ion concentrations in a defined space [53]. Na^+^, K^+^, Ca^2+^, and H^+^ are major intracellular ions [53,54]. In salt-stressed plants, Na^+^ can enter into plant cells, which at high concentrations is harmful to cytosolic enzymes [55]. Therefore, regulation of K^+^ and Na^+^ concentrations to maintain high of K^+^ and low Na^+^ cytosolic levels has a significant impact on salt-stressed plants [54,55]. Restriction of Na^+^ influx, active Na^+^ efflux, and compartmentalization of Na^+^ into the vacuole are three major mechanisms of preventing Na^+^ accumulation in the cytoplasm [56]. The *NHX1* gene encodes a vacuolar Na^+^/H^+^ exchanger, whose homologue in *Arabidopsis*, *AtNHX1,* was upregulated by salt stress resulting in excess transfer of Na^+^ into vacuolar [57]. Salt Overly Sensitive1 (*SOS1*) encodes a transmembrane protein, identified as a plasma membrane Na^+^/H^+^ antiporter. SOS signaling is responsible for transporting Na^+^ out of the cells [37,56]. The *Arabidopsis SOS1* gene possesses 12 transmembrane domains. Similar to *AtNHX1*, *AtSOS1* was also upregulated by salt stress [56]. Besides Na^+^/H^+^ antiporters, the involvement of K^+^ channels has also been reported in plants’ salt stress response. The *AKT1* gene encoding a Shaker type K^+^ channel protein is responsible for absorbing K^+^ from the soil and transporting it into the roots [58]. Under salt stress, *NHX1*, *SOS1* and *AKT1* upregulated gene expression leads to an increase of K^+^ and decreased Na^+^ in plant cells, thereby improving plants’ salt stress tolerance.

Recently, studies have shown that the exogenous application of melatonin improves plants’ ion homeostasis under salt stress. Melatonin significantly increased K^+^ and decreased Na^+^ contents in shoots of maize seedlings, leading to a significantly higher K^+^/Na^+^ ratio in shoots under melatonin-mediated salinity [33]. Improved ion homeostasis may be related to the upregulation of several genes, such as *NHX, SOS* and *AKT*. Under salt stress, *MdNHX1* and *MdAKT1* transcript levels were greatly upregulated by melatonin, which is consistent with the relatively high K^+^ levels and K^+^/Na^+^ ratio in melatonin pretreated *Malus hupehensis* seedlings [21]. Similarly, *NHX1* and *SOS2* expression was higher in melatonin-treated rapeseed seedlings compared to non-treated plants, which correlated with the lower Na^+^/K^+^ ratio [37]. Ca^2+^ signaling plays critical roles in plant biotic and abiotic stress responses; however, no evidence regarding the involvement of Ca^2+^ signaling in melatonin-triggered salinity tolerance exists.

### 2.4. Melatonin Regulates Plant Hormones Metabolism

Plant hormones are important signals for plant growth and development [30]. Melatonin widely participates in the metabolism of most plant hormones, such as indole-3-acetic acid (IAA), abscisic acid (ABA), gibberellic acids (GA), cytokinins (CK), and ethylene [59]. 

The melatonin molecule shares chemical similarities with IAA, both using tryptophan as a substrate in their biosynthesis pathways [60]. It is reported that melatonin acts as a growth regulator and exhibits auxin-like activities [61]. Melatonin promotes vegetative growth and root development in many plant species, such as wheat, barley, rice, *Arabidopsis*, soybean, maize, tomato, etc. [59]. Under stress conditions, the growth-promoting effects of melatonin are higher compared to those in control plants [59]. Melatonin has been proposed to regulate lateral root formation through an IAA-independent pathway in *Arabidopsis* [61]. In contrast, others suggest a certain relationship between melatonin and IAA; for example, a slight increase in endogenous IAA content was observed in *Brassica juncea* [59,62] when treated with exogenous melatonin. Furthermore, application of low concentrations of IAA increases endogenous melatonin levels. At the same time, high concentrations of melatonin inhibit PIN1,3,7 expression and decrease IAA levels in *Arabidopsis* roots, suggesting that melatonin may regulate root growth in *Arabidopsis,* completely or partially, through auxin synthesis and polar auxin transport [60].

Abscisis acid (ABA) and gibberellic acids (GA) are important plant hormones in stress responses. The dynamic balance of endogenous ABA and GA levels is crucial for seed germination [30,63]. Genes related to ABA synthesis—such as *ZEP* and *NCED1*—were upregulated during abiotic stresses, resulting in increased endogenous ABA levels [64]. GA acts as an ABA antagonist [65], and plays essential roles in plant stress tolerance [66]. Studies show that melatonin mediates ABA biosynthesis and metabolism regulation, thus decreasing ABA content under stress conditions. For example, in two drought-stressed *Malus* species, melatonin selectively downregulates *MdNCED3*, a key ABA biosynthesis gene, and upregulates *MdCYP707A1* and *MdCYP707A2,* ABA catabolic genes [67]. Similarly, in perennial ryegrass, exogenous melatonin downregulates ABA biosynthesis genes under heat stress, thereby decreasing ABA content [64]. However, melatonin treatment has no effect on water stress-induced ABA accumulation in maize [68]. Under salt stress, melatonin increased endogenous ABA content in *Elymus nutans*, which was significantly suppressed by fluridone. ABA and fluridone pretreatments had no effect on endogenous melatonin concentration, indicating that ABA might act as a downstream signal that participates in the melatonin-induced cold tolerance. Interestingly, melatonin can also activate the expression of cold-responsive genes to improve plant cold-stress tolerance in an ABA-independent manner. This suggests that both ABA-dependent and ABA-independent pathways might be involved in melatonin-induced cold tolerance [69]. These data suggest that, similar to the heat-related results, under drought and cold stresses, exogenous melatonin can also alleviate salt stress by regulating ABA biosynthesis and catabolism. Under salt stress, *CsNCED1* and *CsNCED2*—ABA synthesis-related genes—transcript levels were reduced in melatonin-pretreated seeds, and genes related to ABA catabolism were significantly increased, thus leading to a decreased ABA content. On the contrary, *GA20ox* and *GA3ox*—genes involved in GA synthesis—were significantly upregulated by melatonin, which is consistent with the increased GA content [30]. Overall, hormone biosynthesis- and catabolism-related research is helpful for understanding melatonin’s mechanisms in response to salt stress.

### 2.5. Melatonin Mediates NO Signaling Pathway

Nitric oxide (NO) is an important messenger and ubiquitous signaling molecule, which participates in various plant physiological processes [70], and responds to abiotic and biotic stresses [41,42,71,72]. In animals, NO is synthesized by NO synthase (NOS) [72], and whether NOS-like proteins exist in plants remains controversial. NOS-like proteins were first identified in plants by Ninnemann and Maier [73]. Initially, *Arabidopsis* nitric oxide associated 1 (*NOA1*) was characterized as a NOS-like gene with NOS activity. However, further research indicated that these proteins function as a GTPases, involved in binding RNA/ribosomes [74]. There are at least seven different NO biosynthetic pathways found in plants, which can be classified as oxidative or reductive based on the operation [75]. Oxidative routes of NO biosynthesis use l-arginine, polyamine, or droxylamine as substrates [75]. S-nitrosylation refers to the process of covalently binding a NO group to its target proteins via cysteine (Cys) residues, and producing an S-nitrosothiol [76]. *S*-nitrosylation, with NO, is widely used to explain NO signaling in both animals and plants [77,78].

Studies have shown that melatonin, through its interaction with NO, plays important roles in plant stress responses. For examples, NO acts as a downstream signal for melatonin mitigated sodic alkaline stress in tomato seedlings [79]. In addition, exogenous melatonin significantly induces the accumulation of polyamine-mediated NO in the roots of *Arabidopsis* under Fe deficiency conditions, and increases the plants’ tolerance to Fe deficiency [80]. Melatonin-induced NO production is also involved in the innate immune response of *Arabidopsis* against P. syringe pv. tomato (Pst) DC3000 infection [81]. In rapeseed seedlings, the possible roles of NO in melatonin-enhanced salt stress tolerance have been reported. Salt stress firstly induces the increase in melatonin and NO serves as the downstream signal. In addition, both melatonin and sodium nitroprusside (SNP) increased salinity-induced S-nitrosylation. Increased S-nitrosylation could be partially impaired by 2-phenyl-1-4,4,5,5-tetramethylimidazoline-1-oxyl-3-oxide (PTIO), an NO scavenger. Application of melatonin increased *NHX1* and *SOS2* transcript levels, which was blocked by NO removal. These data suggest that NO is involved in the maintenance of ion homeostasis in plant salt stress tolerance. NO is also involved in the improvement of the antioxidant systems triggered by melatonin [37]. However, the above research still lacks *S*-nitrosylation target protein identification. In addition, the interactions between NO and other substances, such as hormones, chlorophyll, polyamines, etc., in melatonin-enhanced salt stress tolerance requires further exploration.

### 2.6. Melatonin Regulates Polyamine Metabolism

Polyamines (PAs) are small aliphatic polycations that have been found in almost all living organisms. They play important roles in plant growth and development, and responses to various biotic and abiotic stimuli [82,83,84]. Spermidine (Spd), putrescine (Put), and spermine (Spm) are three main polyamines in plants [84]. Both the application of exogenous polyamines and modulating endogenous polyamine contents effectively enhance plant stress tolerance [83,84].

Studies have shown that melatonin plays a key role in polyamine-mediated signaling pathways under various abiotic stresses, such as alkaline stress, cold, oxidative, and iron deficiency tolerance [7]. For example, polyamines mediate the melatonin-induced alkaline stress tolerance of *Malus hupehensis*. Under alkaline stress, melatonin application significantly upregulated the expression of six polyamine synthesis-related genes, including *SAMDC1*, *-3*, *-4*, and *SPDS1*, *-3*, *-5*, *-6.* Moreover, melatonin-treated *Malus hupehensis* exhibited more polyamine accumulation compared to the untreated seedlings [85]. Exogenous melatonin also modulates polyamine and ABA metabolisms of cucumber seedlings during chilling stress. The melatonin-related cold tolerance improvement is consistent with the increased PA content [24]. PA modulation by melatonin under a salt stress response was also described by Ke et al. [7], where they show that melatonin treatment increases PAs content by accelerating the conversion of arginine and methionine to polyamines in wheat seedlings. At the same time, melatonin suppresses PAO (polyamine oxidase) and DAO (diamine oxidase) activities—two enzymes involved in polyamines metabolism—which decrease melatonin-induced polyamine degradation, thus improving salt stress tolerance [7]. This provides initial evidence that exogenous melatonin treatment enhances plant salt tolerance by regulating PAs, whether the proposed mechanisms are applicable to other plant species requires further investigation. In addition, polyamines are involved in the melatonin-induced NO production in the roots of Fe deficient *Arabidopsis*, and increase the plant tolerance to Fe deficiency [80]. Thus, the interaction between PAs and NO in melatonin-induced salt stress tolerance of plants requires further confirmation.

## 3. Melatonin Correlated Genes and Gene Families in Plants

To further investigate melatonin’s mechanism in regulating salt tolerance in plants, melatonin biosynthesis- and metabolism-related genes, transcription factors, and other related genes and gene families were summarized. 

### 3.1. Putative Genes Involved in Melatonin-Mediated Salt Stress Tolerance

In a wide range of plant species, the melatonin biosynthesis pathway begins with tryptophan, which is converted to tryptamine by tryptophan decarboxylase. Subsequently, tryptamine is converted to serotonin by tryptamine 5-hydroxylase (T5H). In some of the other plant species, the first two steps of the melatonin biosynthesis pathway are reversed. Tryptophan is first converted into 5-hydroxytrytophan by tryptophan 5-hydroxylase (TPH), and then to serotonin by aromatic-l-amino-acid decarboxylase (TDC/AADC) [86]. Although no TPH enzyme been cloned, the presence of ^14^C-5-hydroxytryptophan and ^14^C-serotonin have been detected when using ^14^C-tryptophan as substrate in *Hypericum perforatum* [87]. In the following two steps, three distinct enzymes and two inversed routes were involved. Serotonin N-acetyltransferase (SNAT) catalyzes serotonin into N-acetylserotonin, and N-acetylserotonin was then converted into melatonin by N-acetylserotonin methyl-transferase (ASMT) or caffeic acid O-methyltransferase (COMT). As ASMT/COMT exhibits substrate affinity towards serotonin, and SNAT has substrate affinity toward 5-methoxytryptamine, serotonin could have been first methylated to 5-methoxytryptamine by ASMT/COMT and then to melatonin by SNAT. Different steps involved in the melatonin biosynthesis pathways may occur in different subcellular locations d. In total, six enzymes are involved in plant melatonin biosynthesis, which are related to four different routes. In an *Arabidopsis AtSNAT* mutant, endogenous melatonin content was lower than that in wild-type *Arabidopsis* seedlings. Moreover, the *AtSNAT* mutant was salt hypersensitive compared to wild-type [22]. The possible functions of apple *MzASMT9* were investigated in *Arabidopsis*. Under salt stress, *MzASMT9* transcript levels were upregulated, and melatonin levels were also increased by the ectopic expression of *MzASMT9*, thus leading to an enhanced salt tolerance in transgenic *Arabidopsis* lines [88]. Although there is no direct evidence about the possible roles of TDC, T5H, and COMT in plant salt tolerance, overexpression and suppression of these genes obviously affected endogenous plant melatonin levels [89,90,91,92]. 

The catabolism of phyto-melatonin has also been reported in recent years. Unlike the biosynthesis of melatonin, the metabolism of phyto-melatonin is either through an enzymatic or non-enzymatic pathway [41]. The major melatonin metabolites in plants are *N^1^*-acetyl-*N^2^*-formyl-5-methoxykynuramine (AFMK) and melatonin hydroxylated derivatives, such as 2-hydroxymelatonin and cyclic-3-hydroxymelatonin (3-OHM) [41,93,94].

In rice, melatonin is catabolized into 2-hydroxymelatonin by melatonin 2-hydroxylase (M2H), which belongs to the 2-oxoglutarate-dependent dioxygenase (2-ODD) superfamily [95]. The first *M2H* gene was cloned from rice in 2015 [96].

Except for genes involved in the biosynthesis and catabolism of phyto-melatonin, transcription factors also play critical roles in the melatonin-mediated salt stress response. Under abiotic stress (salt, drought, and cold), exogenous melatonin significantly improves endogenous melatonin levels and upregulates the expression of C-repeat binding factors (CBFs)/Drought response element Binding 1 factors (DREB1s), thus leading to an increase in transcript levels of multiple stress-responsive genes, including *COR15A*, *RD22,* and *KIN1* [23]. RNA sequencing was performed in cucumber roots with or without melatonin treatment under salt stress. The results show that many transcription factors including WRKY, MYB, NAC, and the ethylene-responsive transcription factor were differentially expressed in melatonin-treated plants compared to control plants under NaCl-induced stress [97].

The effects of melatonin on the expression of genes involved in ROS scavenging under NaCl stress were investigated. The application of 1 mM melatonin induced the expression of *CsCu-ZnSOD*, *CsFe-ZnSOD*, *CsPOD*, and *CsCAT* in cucumber under salt stress [30]. Similar results were also observed in rapeseed, and studies showed that antioxidant defense-related genes such as *APX*, *Cu/ZnSOD* and *MnSOD* were involved in melatonin-induced salt stress tolerance [37]. In tomato seedlings under salt stress, melatonin significantly improved *TRXf* gene expression, which participates in the redox regulation of many physiological processes [44]. Genes responsible for maintaining ion homeostasis were also involved in melatonin-enhanced salt stress. *MdNHX1* and *MdAKT1*, two ion-channel genes, were upregulated by exogenous melatonin in *Malus hupehensis* under salinity [21]. *NHX1* and *SOS2 expression* was also modulated by melatonin in salt-stressed rapeseed. Several studies have shown that melatonin alleviates salinity stress by regulating hormone biosynthesis and metabolism gene expression. Melatonin induced the expression of GA biosynthesis genes (*GA20ox* and *GA3ox*). Meanwhile, the ABA catabolism genes, *CsCYP707A1* and *CsCYP707A2,* were obviously upregulated, whereas the ABA biosynthesis gene *CsNECD2* was downregulated by melatonin in salt-stressed cucumber seedlings [30]. 

### 3.2. Comparative and Phylogenetic Analysis of TDC, T5H, SNAT, and ASMT Gene Families in Plants

*TDC*, *T5H*, *SNAT*, and *ASMT* correlate with melatonin biosynthesis in most plant species [86]. Recently, a genome-wide expression, classification, phylogenetic, and expression profiles of the tryptophan decarboxylase (TDC) gene family was conducted in *Solanum lycopersicum* [98]. A total of five *TDC* genes were obtained from the tomato genome. Among the five candidate genes, *SlTDC3* was expressed in all the tested tissues, whereas *SlTDC1* and *SlTDC2* were specifically expressed in the fruit and leaves of the tomato plant, respectively. *SlTDC4* and *SlTDC5* are not expressed in tomato. The study of *TDC* genes in rice is relatively clearer compared to other plants. Rice has at least three *TDC* genes [89]. *OsTDC1* (AK31) and *OsTDC2* (AK53) were first identified by Kang et al. [99]. Heterologous expression of *OsTDC1* and *OsTDC2* in *Escherichia coli* showed that both genes exhibited TDC activity [99]. The expression profiles of *OsTDC1*, *OsTDC2*, and *OsTDC3* have also been investigated in rice. *OsTDC1* and *OsTDC2* have similar expression profiles, with low expression in seedling shoots, and relatively high levels in leafs, stems, roots and flowers. In comparison, *OsTDC3* expression was very low in almost all tested organs, except the roots [89]. These results indicated that different *TDC* genes might play different roles during plant growth and development. Overexpression of *OsTDC1*, *OsTDC2,* and especially *OsTDC3* leads to improved melatonin levels in transgenic rice [89]. The phylogenetic relationships and gene structures of TDCs from algae to higher plants showed that they are found throughout the high plant kingdom with a small family size. The evolution of *TDC* genes in plants was mainly through gene expansion and intron loss events. This is the first research of its kind on the TDC gene family; however, the expression profiles of TDCs were not investigated under the salt stress condition [98,99,100]. The ASMT gene family was also analyzed in *Solanum lycopersicum* [101]. There are 14 candidate *ASMT* genes involved in tomato, three of which may be pseudogenes. The expression patterns of *SlASMT*s suggested that four *SlASMT*s were involved in tomato plant response to biotic stresses [101].

**Figure 1:**
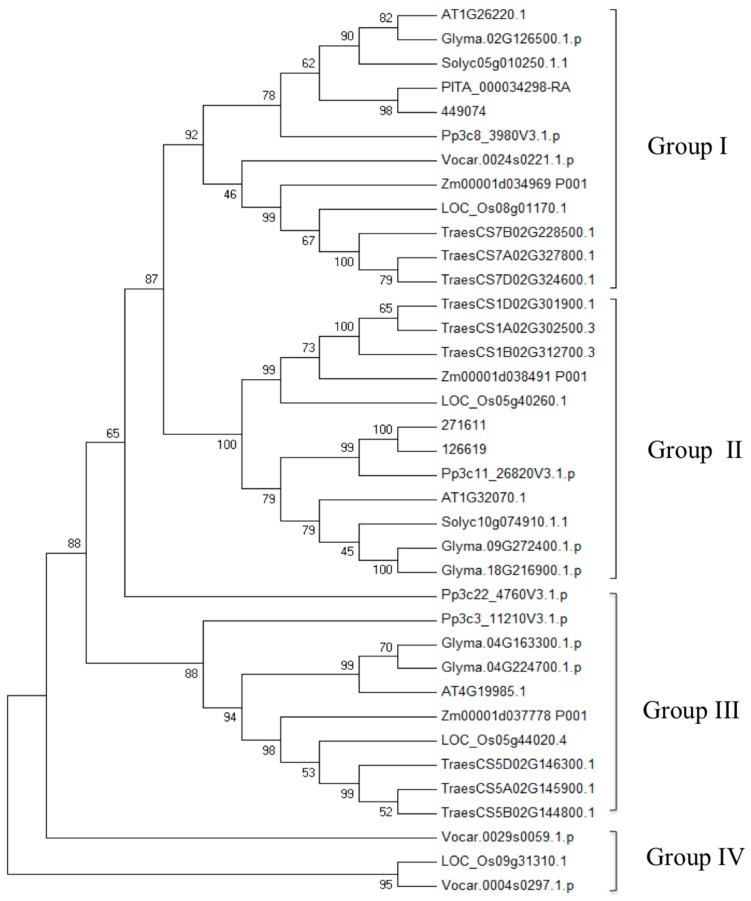
Phylogenetic relationship of the serotonin N-acetyltransferase (*SNAT*) genes from 10 plant species. The candidate *SNAT* genes involved in the phylogenetic tree include the dicots (*Arabidopsis.thaliana* (AT): AT1G26220.1, AT1G32070.1, and AT4G19985.1; *Solanum lycopersicum* (Solyc): Solyc05g010250.1.1, and Solyc10g074910.1.1; *Glyma max* (Glyma): Glyma.02G126500.1.p, Glyma.04G163300.1.p, Glyma.04G224700.1.p, Glyma.09G272400.1.p, and Glyma.18G216900.1.p), monocot (*Zea mays* (Zm): Zm00001d037778_P001, Zm00001d034969_P001, and Zm00001d038491_P001; *Oryza sativa* (LOC_Os): LOC_Os05g40260.1, LOC_Os05g44020.4, LOC_Os08g01170.1, and LOC_Os09g31310.1; *Triticum aestivum* (Traes): TraesCS5D02G146300.1, TraesCS7B02G228500.1, TraesCS7A02G327800.1, TraesCS7D02G324600.1, TraesCS5A02G145900.1, TraesCS1D02G301900.1, TraesCS5B02G144800.1, TraesCS1B02G312700.3, and TraesCS1A02G302500.3), Gymnospermae (*Pinus taeda* (PITA): PITA_000034298-RA), Pteridophyta (*Selaginella moellendorffii*: 271611, 449074, and 126619), Bryophyta (*Physcomitrella patens* (Pp): Pp3c22_4760V3.1.p, Pp3c3_11210V3.1.p, Pp3c11_26820V3.1.p, and Pp3c8_3980V3.1.p), and algae (*Volvox carteri* (Vocar): Vocar.0029s0059.1.p, Vocar.0004s0297.1.p, and Vocar.0024s0221.1.p)

**Figure 2:**
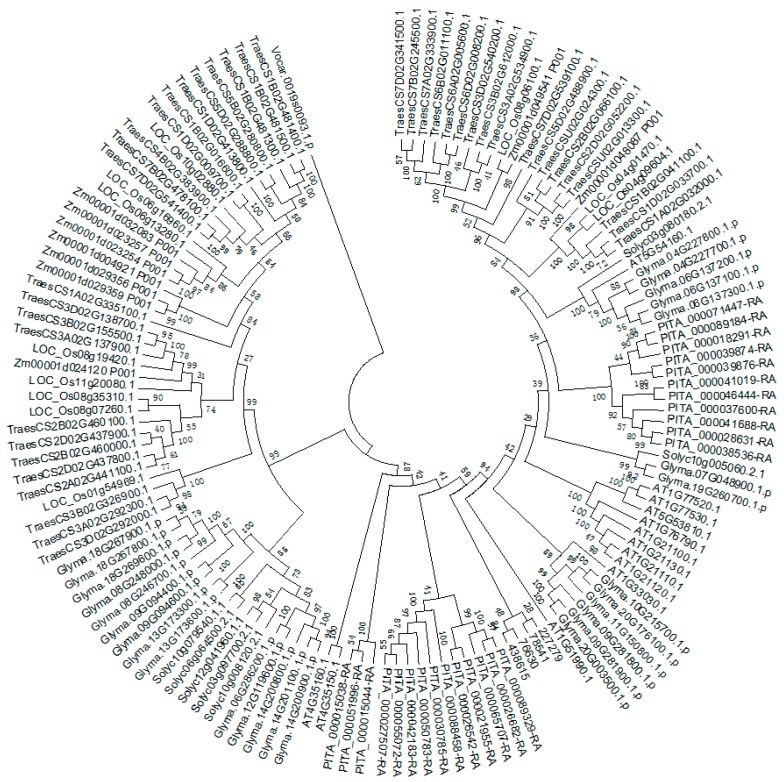
Phylogenetic relationship of the N-acetylserotonin methyl-transferase (*ASMT*) genes from 10 plant species. The 10 plant species include *A.thaliana*, *S.lycopersicum*, *G.max*, *Z.mays*, *O.sativa*, *T.aestivum*, *P.taeda*, *S.moellendorffii*, *P.patens,* and algae.

*TDC*, *T5H*, *SNAT*, and genes have been identified and functionally analyzed in many plants, especially in rice [89,91,102,103,104]. A systematic analysis of the tomato TDC gene family has been conducted, and the phylogenetic relationships between *TDC* genes in plants have also been analyzed. In addition to the *ASMT* gene families in tomato, genome-wide analysis of *SNAT*, *ASMT*, and *T5H* families has not been reported. Based on the methods described by Pang et al. [98] and Liu et al. [101], we searched *TDC* genes in wheat genome, as well as *SNAT* and *ASMT* genes in 10 plant species from algae to higher plants. We further validated these *TDC* and *ASMT* genes using the previously reported main residues [105,106,107]. Only BLASTP (identity >70%, coverage >70%) was conducted for *T5H* genes identification, using rice *T5H* genes as the query. A total of eight *T5H*, 37 *SNAT*, and 140 *ASMT* candidate genes were obtained in 10 plant species (Supplementary Table S1). Furthermore, there are 33 candidate *TDC* genes in wheat. Phylogenetic relationships of *SNAT* and *ASMT* are shown in Figure 1 and Figure 2. Based on the phylogenetic tree topology, the SNAT gene family could be divided into four groups (Group I to IV). *SNAT* members in Group I are highly conserved across all species. Similar numbers of *SNAT* genes were found in different species, and no obvious gene expansion was observed. *OsSNAT2* of rice belongs to Group I, whose function is already revealed [108]. The *ASMT* gene family phylogenetic tree is similar to that of the *TDC* gene family [100]. One member from *Volvox carteri* clustered into a separate branch, indicating that *ASMT* genes originated before the divergence of green algae and land plant species. The average gene number of *ASMT* in algae, pteridophyta, gymnosperms, and angiosperms is 1, 4, 25, and 18.3, respectively, suggesting that gene expansion occurred during the evolution from algae to higher plants.

**Figure 3:**
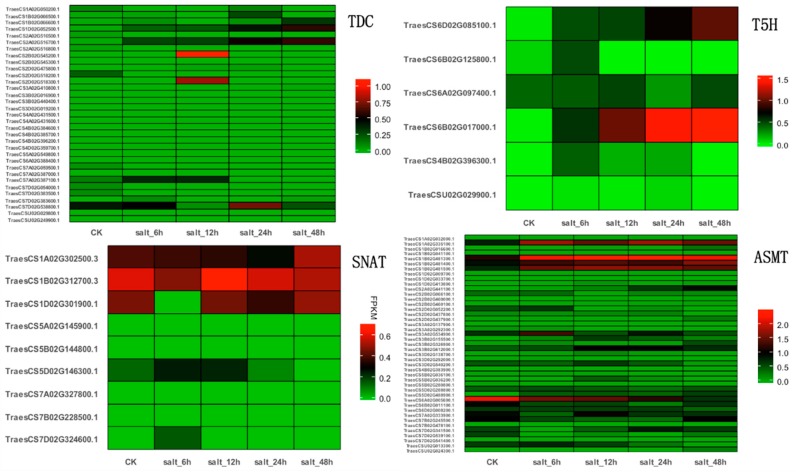
Expression profiles of *TDC*, *T5H*, *SNAT*, and *ASMT* genes in wheat under salt stress conditions. The red or green colors represent the higher or lower relative abundance of each transcript in each sample, respectively.

Furthermore, we specially investigated the expression profiles of *TDC*, *T5H*, *SNAT*, and *ASMT* genes in wheat under salt stress. RNA-sequencing data were downloaded from the NCBI Sequence Read Archive (SRA) ddabase (https://www.ncbi.nlm.nih.gov/sra/). FPKM (fragments per kilobase of transcript per million fragments mapped) values for all candidate genes in wheat were calculated using Hisat2 and Stringtie, and the heat maps were generated using the geom_tile method in ggplot2 [109]. As shown in Figure 3, there are four *TDC* genes, two *T5H* genes, one *SNAT* gene, and 10 *ASMT* genes specifically expressed under salt stress, and lots of genes are upregulated under salt conditions, indicating that these genes could be involved in the salt stress tolerance of wheat.

## 4. Phyto-Melatonin Receptor

It is clear that exogenous melatonin plays a considerable role during plant growth and development, and is associated with plant stress responses—including salt stress. However, the method by which plants perceive exogenous melatonin and convert it into downstream signals remains unknown. The phyto-melatonin receptor holds promise for better understanding melatonin’s biological function and mechanism. Animal melatonin receptors were discovered earlier than the phyto-melatonin receptor. The first melatonin receptor (Mel1c) was cloned from frogs (*Xenopus laevis*) in 1994 [110]. Melatonin receptors belong to the G protein-coupled receptor (GPCR) superfamily, which possess seven transmembrane helices [111]. To date, a total of three melatonin receptor subtyoes have been reported in mammals; MT1 (Mel1a), MT2 (Mel1b), and MT3 (ML2) [112,113]. MT1 and MT2 are G protein-coupled receptors, which exhibit high-affinity for melatonin [112,114], while MT3 exhibits low affinity for melatonin and it belongs to the quinone reductases family [115]. 

*AtCAND2/PMTR1*, the first phyto-melatonin receptor, was recently discovered in *Arabidopsis*. When melatonin is perceived by CAND2/PMTR1, it triggers the dissociation of Gα form Gγβ, which activates the downstream H_2_O_2_ and Ca^2+^ signaling transduction cascade, leading to the phenotype of stomatal closure. Several studies have identified CAND2 as the first phyto-melatonin receptor. *AtCAND2* is a membrane protein with seven transmembrane helices. Interaction with the unique G proteinαsubunit (GPA1) of *Arabidopsis* proved that CAND2 is a G protein-coupled receptor. ^125^I-melatonin can bind to CAND2 in a specific and saturated manner. *Arabidopsis AtCand2* mutant exhibits no changes in the stomatal aperture when treated with melatonin, while 10 μmol/L melatonin induced stomatal closure in the wild-type counterparts [114]. These data indicate that further research on CAND2/PMTR1-mediated signaling in salt stress is required. Moreover, the discovery of CAND2/PMTR1 provides a new method for finding other melatonin receptors in plants.

## 5. Conclusions and Future Perspectives

**Figure 4:**
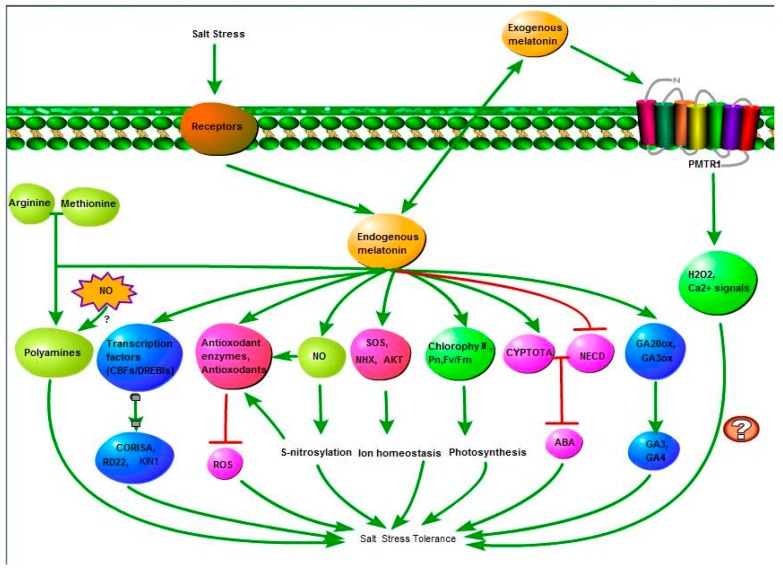
Melatonin-mediated salt stress response in plants. Abbreviation: NO, nitric oxide; ROS, reactive oxygen species; Pn, net photosynthetic rate; ABA, abscisic acid; GA, gibberellin acid. ⊥: represents inhibition; and →: represents promotion.

Melatonin, as an antioxidant and signaling molecule, modulates a wide range of physiological functions in bacteria, fungi, invertebrates, vertebrates, algae, and plants. It has been extensively studied in humans and other animals, while plant studies have lagged behind. In light of its importance and significance, more and more attention has focused on the biosynthesis and bio-function of melatonin in plants. It has become a research hotspot in the plant biology kingdom, with increasing research being conducted in recent years [116,117]. To promote related research in plant salt tolerance, we summarized the regulatory roles and mechanisms of melatonin in plants during salt stress resistance by reviewing recently published literature, and we finally propose a model (Figure 4).

First, salt stress or the application of exogenous melatonin improves endogenous melatonin levels in plants, which modulates the expression of genes involved in melatonin biosynthesis and metabolisms or assimilates exogenous melatonin directly [116]. Increased levels of endogenous melatonin occur mainly by upregulation of melatonin biosynthesis-related genes or absorption of exogenous melatonin by plants; both mechanisms require further investigation. Increased endogenous levels enhanced plant salt stress tolerance via several different pathways. The improvement of antioxidant capacity, ion homeostasis, photosynthetic capacity and the regulation of ROS, NO, hormone, and polyamine metabolism by melatonin in salt-stressed plants was discussed. Previous studies have shown that Ca^2+^ signaling plays important roles in salt stress tolerance [118]; however, little evidence of Ca^2+^ signaling was observed in the melatonin-induced salt stress tolerance. Therefore, whether melatonin enhances plants salinity resistance through Ca^2+^ signaling requires further investigation.

Genetic modification and RNA-sequencing analysis are effective tools in the identification of the putative target genes involved in melatonin-enhanced salt stress tolerance. TPH, a putative gene involved in serotonin biosynthesis, has not been cloned in plants yet. However, *TDC* and *T5H*, two genes involved in serotonin biosynthesis, have been identified in many plants, but have not been cloned in *Arabidopsis*. We suspect that other biosynthesis pathways of melatonin may also exist in plants. 

Plant melatonin receptors have been the bottleneck in the study of phyto-melatonin in the past few decades. With the first phytomelatonin receptor discovered recently in *Arabidopsis*, the involvement of PMTR1-mediated phytomelatonin signaling in salt stress response requires updated exploration. In addition, three melatonin receptors MT1, MT2, and MT3, have been identified in mammals, the identification of new phytomelatonin receptors is another exciting field to explore. Further studies in this field might deepen our understanding of the biological functions and molecular mechanisms governing melatonin’s regulatory role during salt stress tolerance and beyond. 

## Figures and Tables

**Table 1:** The reported roles melatonin plays in response to salt and other stresses in plants.

Plant Species	Stress Condition	References
*Actinidia deliciosa*	Salt	[20]
*Malus hupehensis*	Salt	[21]
*Arabidopsis thaliana*	salt	[22]
*Arabidopsis thaliana*	Salt, drought and cold	[23]
*Arabidopsis thaliana*	Salt	[24]
*Cynodon dactylon* (L). Pers.	Salt, drought and cold	[25]
*Chara australis*	Salt	[26]
*Chlamydomonas reinhardtii*	Salt	[27]
*Citrus aurantium* L.	Salt	[28]
*Cucumis sativus* L.	Salt	[29]
*Cucumis sativus* L.	Salt	[17]
*Cucumis sativus* L.	Salt	[30]
*Zea mays* L.	Salt	[31]
*Zea mays* L.	Salt	[32]
*Zea mays* L.	Salt	[33]
*Raphanus sativus* L.	Salt	[34]
*Raphanus sativus* L.	Salt	[35]
*Brassica napus* L.	Salt	[36]
*Brassica napus* L.	Salt	[37]
*Oryza sativa* L.	Leaf senescence and salt	[38]
*Oryza sativa* L.	Salt	[39]
*Glycine max*	Salt and drought	[40]
*Helianthus annuus*	Salt	[41]
*Helianthus annuus*	Salt	[42]
*Ipomoea batatas*	Salt	[43]
*Solanum lycopersicum*	Salt	[44]
*Vicia faba* L.	Salt	[45]
*Citrullus lanatus* L.	Salt	[46]
*Triticum aestivum* L.	Salt	[47]

## Supplementary Materials

Supplementary materials can be found at http://www.mdpi.com/1422-0067/20/3/709/s1.

## References

[1] Munns R., Tester M. (2008). Mechanisms of salinity tolerance. Annu. Rev. Plant Biol..

[2] Abbasi H., Jamil M., Haq A., Ali S., Ahmad R., Malik Z., Parveen (2016). Salt stress manifestation on plants, mechanism of salt tolerance and potassium role in alleviating it: A review. Zemdirbyste-Agriculture.

[3] Rahnama A., James R., Poustini K., Munns R. (2010). Stomatal conductance as a screen for osmotic stress tolerance in durum wheat growing in saline soil. Funct. Plant Biol..

[4] Ashraf M., Wu L. (1994). Breeding for salinity tolerance in plants. Crit. Rev. Plant Sci..

[5] Shalata A., Mittova V., Volokita M., Guy M., Tal M. (2001). Response of the cultivated tomato and its wild salt-tolerant relative Lycopersicon pennellii to salt-dependent oxidative stress: The root antioxidative system. Physiol. Plant.

[6] Hasanuzzaman M., Oku H., Nahar K., Bhuyan M.H.M.B., Mahmud J.A., Baluska F., Fujita M. (2018). Nitric oxide-induced salt stress tolerance in plants: ROS metabolism, signaling, and molecular interactions. Plant Biotechnol. Rep..

[7] Ke Q., Ye J., Wang B., Ren J., Yin L., Deng X., Wang S. (2018). Melatonin mitigates salt stress in wheat seedlings by modulating polyamine metabolism. Front. Plant Sci..

[8] Lerner A.B., Case J.D., Takahashi Y., Lee T.H., Mori W. (1958). Isolation of melatonin, the pineal gland factor that lightens melanocyteS1. J. Am. Chem. Soc..

[9] Brainard G.C., Hanifin J.P., Greeson J.M., Byrne B., Glickman G., Gerner E., Rollag M.D. (2001). Action spectrum for melatonin regulation in humans: evidence for a novel circadian photoreceptor. J. Neurosci..

[10] Mishima K. (2012). Melatonin as a regulator of human sleep and circadian systems. Nihon Rinsho.

[11] Rodriguez C., Mayo J.C., Sainz R.M., Antolín I., Herrera F., Martín V., Reiter R.J. (2004). Regulation of antioxidant enzymes: a significant role for melatonin. J. Pineal Res..

[12] Calvo J.R., González-Yanes C., Maldonado M.D. (2013). The role of melatonin in the cells of the innate immunity: a review. J. Pineal Res..

[13] Barrett P., Bolborea M. (2012). Molecular pathways involved in seasonal body weight and reproductive responses governed by melatonin. J. Pineal Res..

[14] Dollins A.B., Zhdanova I.V., Wurtman R.J., Lynch H.J., Deng M.H. (1994). Effect of inducing nocturnal serum melatonin concentrations in daytime on sleep, mood, body temperature, and performance. Proc. Natl. Acad. Sci. USA.

[15] Hattori A., Migitaka H., Iigo M., Itoh M., Yamamoto K., Ohtani-Kaneko R., Hara M., Suzuki T., Reiter R.J. (1995). Identification of melatonin in plants and its effects on plasma melatonin levels and binding to melatonin receptors in vertebrates. Biochem. Mol. Biol. Int..

[16] Dubbels R., Reiter R.J., Klenke E., Goebel A., Schnakenberg E., Ehlers C., Schiwara H.W., Schloot W. (1995). Melatonin in edible plants identified by radioimmunoassay and by high performance liquid chromatography-mass spectrometry. J. Pineal Res..

[17] Wang L.Y., Liu J.L., Wang W.X., Sun Y. (2016). Exogenous melatonin improves growth and photosynthetic capacity of cucumber under salinity-induced stress. Photosynthetica.

[18] Tan D.-X., Hardeland R., Manchester L.C., Korkmaz A., Ma S., Rosales-Corral S., Reiter R.J. (2012). Functional roles of melatonin in plants, and perspectives in nutritional and agricultural science. J. Exp. Bot..

[19] Yu Y., Lv Y., Shi Y., Li T., Chen Y., Zhao D., Zhao Z. (2018). The Role of Phyto-Melatonin and Related Metabolites in Response to Stress. Molecules.

[20] Xia H., Ni Z., Pan D. (2017). Effects of exogenous melatonin on antioxidant capacity in Actinidia seedlings under salt stress. IOP Conf. Ser. Earth Environ. Sci..

[21] Li C., Wang P., Wei Z., Liang D., Liu C., Yin L., Jia D., Fu M., Ma F. (2012). The mitigation effects of exogenous melatonin on salinity-induced stress in Malus hupehensis. J. Pineal Res..

[22] Chen Z., Xie Y., Gu Q., Zhao G., Zhang Y., Cui W., Xu S., Wang R., Shen W. (2017). The AtrbohF-dependent regulation of ROS signaling is required for melatonin-induced salinity tolerance in Arabidopsis. Free Radical Bio. Med..

[23] Shi H., Qian Y., Tan D.-X., Reiter R.J., He C. (2015). Melatonin induces the transcripts of CBF/DREB1s and their involvement in both abiotic and biotic stresses in Arabidopsis. J. Pineal Res..

[24] Zheng X., Tan D.X., Allan A.C., Zuo B., Zhao Y., Reiter R.J., Wang L., Wang Z., Guo Y., Zhou J. (2017). Chloroplastic biosynthesis of melatonin and its involvement in protection of plants from salt stress. Sci. Rep..

[25] Shi H., Jiang C., Ye T., Tan D.-x., Reiter R.J., Zhang H., Liu R., Chan Z. (2015). Comparative physiological, metabolomic, and transcriptomic analyses reveal mechanisms of improved abiotic stress resistance in bermudagrass (*Cynodon dactylon* (L). Pers.) by exogenous melatonin. J. Exp. Bot..

[26] Beilby M.J., Al Khazaaly S., Bisson M.A. (2015). Salinity-induced noise in membrane potential of Characeae chara australis: effect of exogenous melatonin. J. Membrane Biol..

[27] Zhang Y., Gao W., Lv Y., Bai Q., Wang Y. (2018). Exogenous melatonin confers salt stress tolerance to Chlamydomonas reinhardtii (Volvocales, Chlorophyceae) by improving redox homeostasis. Phycologia.

[28] Kostopoulou Z., Therios I., Roumeliotis E., Kanellis A.K., Molassiotis A. (2015). Melatonin combined with ascorbic acid provides salt adaptation in *Citrus aurantium* L. seedlings. Plant Physiol. Bioch..

[29] Zhang N., Zhang H.-J., Sun Q.-Q., Cao Y.-Y., Li X., Zhao B., Wu P., Guo Y.-D. (2017). Proteomic analysis reveals a role of melatonin in promoting cucumber seed germination under high salinity by regulating energy production. Scientific reports.

[30] Zhang H.-J., Zhang N., Yang R.-C., Wang L., Sun Q.-Q., Li D.-B., Cao Y.-Y., Weeda S., Zhao B., Ren S. (2014). Melatonin promotes seed germination under high salinity by regulating antioxidant systems, ABA and GA4 interaction in cucumber (*Cucumis sativus* L.). J. Pineal Res..

[31] Chen Y.-E., Mao J.-J., Sun L.-Q., Huang B., Ding C.-B., Gu Y., Liao J.-Q., Hu C., Zhang Z.-W., Yuan S. (2018). Exogenous melatonin enhances salt stress tolerance in maize seedlings by improving antioxidant and photosynthetic capacity. Physiol. Plant.

[32] Jiang X., Li H., Song X. (2016). Seed priming with melatonin effects on seed germination and seedling growth in maize under salinity stress. Pak. J. Bot..

[33] Jiang C., Cui Q., Feng K., Xu D., Li C., Zheng Q. (2016). Melatonin improves antioxidant capacity and ion homeostasis and enhances salt tolerance in maize seedlings. Acta Physiol. Plant..

[34] Jiang Y., Liang D., Liao M.A., Lin L. Effects of melatonin on the growth of radish Seedlings under salt stress. Proceedings of the 3rd international conference on renewable energy and environmental technology (ICERE 2017).

[35] Yao H., Wang X., Liao M.A., Lin L. Effects of melatonin treated radish on the growth of following stubble lettuce under salt stress. Proceedings of the 3rd international conference on renewable energy and environmental technology (ICERE 2017).

[36] Zeng L., Cai J.S., Li J.J., Lu G.Y., Li C.S., Fu G.P., Zhang X.K., Ma H.Q., Liu Q.Y., Zou X.L. (2018). Exogenous application of a low concentration of melatonin enhances salt tolerance in rapeseed (Brassica napus L.) seedlings. J. Integ. Agr.

[37] Zhao G., Zhao Y., Yu X., Kiprotich F., Han H., Guan R., Wang R., Shen W. (2018). Nitric oxide is required for melatonin-enhanced tolerance against salinity stress in rapeseed (*Brassica napus* L.) seedlings. Int. J. MolSci..

[38] Liang C., Zheng G., Li W., Wang Y., Hu B., Wang H., Wu H., Qian Y., Zhu X.-G., Tan D.-X. (2015). Melatonin delays leaf senescence and enhances salt stress tolerance in rice. J. Pineal Res..

[39] Li X., Yu B., Cui Y., Yin Y. (2017). Melatonin application confers enhanced salt tolerance by regulating Na+ and Cl− accumulation in rice. Plant Growth Regul..

[40] Wei W., Li Q.-T., Chu Y.-N., Reiter R.J., Yu X.-M., Zhu D.-H., Zhang W.-K., Ma B., Lin Q., Zhang J.-S. (2015). Melatonin enhances plant growth and abiotic stress tolerance in soybean plants. J. Exp Bot..

[41] Arora D., Bhatla S.C. (2017). Melatonin and nitric oxide regulate sunflower seedling growth under salt stress accompanying differential expression of Cu/Zn SOD and Mn SOD. Free Radical Biol. Med..

[42] Kaur H., Bhatla S.C. (2016). Melatonin and nitric oxide modulate glutathione content and glutathione reductase activity in sunflower seedling cotyledons accompanying salt stress. Nitric Oxide.

[43] Yu Y., Wang A., Li X., Kou M., Wang W., Chen X., Xu T., Zhu M., Ma D., Li Z. (2018). Melatonin-stimulated triacylglycerol breakdown and energy turnover under salinity stress contributes to the maintenance of plasma membrane H^+^-ATPase activity and K^+^/Na^+^ homeostasis in sweet potato. Front. Plant Sci..

[44] Zhou X., Zhao H., Cao K., Hu L., Du T., Baluška F., Zou Z. (2016). Beneficial roles of melatonin on redox regulation of photosynthetic electron transport and synthesis of D1 protein in tomato seedlings under salt stress. Front. Plant Sci..

[45] Dawood M.G., El-Awadi M.E. (2015). Alleviation of salinity stress on *Vicia faba* L. plants via seed priming with melatonin. Acta Biológica Colombiana.

[46] Li H., Chang J., Chen H., Wang Z., Gu X., Wei C., Zhang Y., Ma J., Yang J., Zhang X. (2017). Exogenous Melatonin Confers Salt Stress Tolerance to Watermelon by Improving Photosynthesis and Redox Homeostasis. Front. Plant Sci..

[47] El-Mashad A.A.A., Mohamed H.I. (2012). Brassinolide alleviates salt stress and increases antioxidant activity of cowpea plants (*Vigna sinensis*). Protoplasma.

[48] Zhang M., Smith J.A.C., Harberd N.P., Jiang C. (2016). The regulatory roles of ethylene and reactive oxygen species (ROS) in plant salt stress responses. Plant Mol. Biol..

[49] Ahmad P., Abdul Jaleel C., A Salem M., Nabi G., Sharma S. (2010). Roles of Enzymatic and non-enzymatic antioxidants in plants during abiotic stress. Crit Rev Biotechnol..

[50] Tan D.X., Manchester L.C., Terron M.P., Flores L.J., Reiter R.J. (2007). One molecule, many derivatives: A never-ending interaction of melatonin with reactive oxygen and nitrogen species?. J. Pineal Res..

[51] Campos L.M.O., Hsie S.B., Granja A.J.A., Correia M.R., Almeida-Cortez J., Pompelli M.F. (2012). Photosynthesis and antioxidant activity in *Jatropha curcas* L. under salt stress. Braz. J. Plant Physiol..

[52] Meloni D.A., Oliva M.A., Martinez C.A., Cambraia J. (2003). Photosynthesis and activity of superoxide dismutase, peroxidase and glutathione reductase in cotton under salt stress. Environ. Exp. Bot..

[53] Amtmann A., Leigh R., Pareek A., Sopory S.K., Bohnert H.J. (2010). Ion Homeostasis. Abiotic Stress Adaptation in Plants: Physiological, Molecular and Genomic Foundation.

[54] Zhu J.K. (2003). Regulation of ion homeostasis under salt stress. Curr. Opin. Plant Biol..

[55] Fukuda A., Nakamura A., Hara N., Toki S., Tanaka Y. (2011). Molecular and functional analyses of rice NHX-type Na^+^/H^+^ antiporter genes. Planta.

[56] Padan E., Venturi M., Gerchman Y., Dover N. (2001). Na^+^/H^+^ antiporters. BBA- Bioenergetics.

[57] Shi H., Zhu J.-K. (2002). Regulation of expression of the vacuolar Na^+^/H^+^ antiporter gene AtNHX1 by salt stress and abscisic acid. Plant Mol. Biol..

[58] Garriga M., Raddatz N., Véry A.-A., Sentenac H., Rubio-Meléndez M.E., González W., Dreyer I. (2017). Cloning and functional characterization of HKT1 and AKT1 genes of *Fragaria* spp.—Relationship to plant response to salt stress. J. Plant. Physiol..

[59] Arnao M.B., Hernández-Ruiz J. (2018). Melatonin and its relationship to plant hormones. Ann. Bot.

[60] Wang Q., An B., Wei Y., Reiter R.J., Shi H., Luo H., He C. (2016). Melatonin regulates root meristem by repressing auxin synthesis and polar auxin transport in Arabidopsis. Front. Plant Sci..

[61] Pelagio-Flores R., Muñoz-Parra E., Ortiz-Castro R., López-Bucio J. (2012). Melatonin regulates Arabidopsis root system architecture likely acting independently of auxin signaling. J. Pineal Res..

[62] Chen Q., Qi W.-b., Reiter R.J., Wei W., Wang B.-m. (2009). Exogenously applied melatonin stimulates root growth and raises endogenous indoleacetic acid in roots of etiolated seedlings of Brassica juncea. J. Plant Physiol..

[63] Footitt S., Douterelo-Soler I., Clay H., Finch-Savage W.E. (2011). Dormancy cycling in Arabidopsis seeds is controlled by seasonally distinct hormone-signaling pathways. Proc. Natl. Acad. Sci. USA.

[64] Zhang J., Shi Y., Zhang X., Du H., Xu B., Huang B. (2017). Melatonin suppression of heat-induced leaf senescence involves changes in abscisic acid and cytokinin biosynthesis and signaling pathways in perennial ryegrass (*Lolium perenne* L.). Environ. Exp. Bot..

[65] Yang R., Yang T., Zhang H., Qi Y., Xing Y., Zhang N., Li R., Weeda S., Ren S., Ouyang B. (2014). Hormone profiling and transcription analysis reveal a major role of ABA in tomato salt tolerance. Plant Physiol. Bioch..

[66] Maggio A., Barbieri G., Raimondi G., De Pascale S. (2010). Contrasting Effects of GA3 Treatments on Tomato Plants Exposed to Increasing Salinity. J. Plant Growth Regul..

[67] Li C., Tan D.-X., Liang D., Chang C., Jia D., Ma F. (2015). Melatonin mediates the regulation of ABA metabolism, free-radical scavenging, and stomatal behaviour in two Malus species under drought stress. J. Exp. Bot..

[68] Jia W., Zhang J. (2000). Water stress-induced abscisis acid accumulation in relation to reducing agents and sulfhydryl modifiers in maize plant. Plant Cell Environ..

[69] Fu J., Wu Y., Miao Y., Xu Y., Zhao E., Wang J., Sun H., Liu Q., Xue Y., Xu Y. (2017). Improved cold tolerance in Elymus nutans by exogenous application of melatonin may involve ABA-dependent and ABA-independent pathways. Scientific Reports.

[70] Aydogan S., Yerer M.B., Goktas A. (2006). Melatonin and nitric oxide. J. Endocrinol. Invest..

[71] Zhao M.G., Tian Q.Y., Zhang W.H. (2007). Nitric oxide synthase-dependent nitric oxide production is associated with salt tolerance in Arabidopsis. Plant Physiol..

[72] Lozano-Juste J., León J. (2010). Enhanced abscisic acid-mediated responses in *nia1nia2noa1-2* triple mutant impaired in NIA/NR- and *AtNOA1*-dependent nitric oxide biosynthesis in Arabidopsis. Plant Physiol..

[73] Ninnemann H., Maier J. (1996). Indications for the occurrence of nitric oxide synthases in fungi and plants and the involvement in photoconidiation of *Neurospora crassa*. Photochem. Photobiol..

[74] Corpas F.J., Palma J.M., Del Río L.A., Barroso J.B. (2009). Evidence supporting the existence of l-arginine-dependent nitric oxide synthase activity in plants. New Phytologist.

[75] Gupta K.J., Fernie A.R., Kaiser W.M., van Dongen J.T. (2011). On the origins of nitric oxide. Trends Plant Sci..

[76] Astier J., Rasul S., Koen E., Manzoor H., Besson-Bard A., Lamotte O., Jeandroz S., Durner J., Lindermayr C., Wendehenne D. (2011). S-nitrosylation: An emerging post-translational protein modification in plants. Plant Sci..

[77] Gupta K.J. (2011). Protein S-nitrosylation in plants: photorespiratory metabolism and NO signaling. Sci Signal..

[78] Jaffrey S.R., Erdjument-Bromage H., Ferris C.D., Tempst P., Snyder S.H. (2001). Protein S-nitrosylation: A physiological signal for neuronal nitric oxide. Nat. Cell Biol..

[79] Liu N., Gong B., Jin Z., Wang X., Wei M., Yang F., Li Y., Shi Q. (2015). Sodic alkaline stress mitigation by exogenous melatonin in tomato needs nitric oxide as a downstream signal. J. Plant Physiol..

[80] Zhou C., Liu Z., Zhu L., Ma Z., Wang J., Zhu J. (2016). Exogenous melatonin improves plant iron deficiency tolerance via increased accumulation of polyamine-mediated nitric oxide. Int. J. Mol. Sci..

[81] Shi H., Chen Y., Tan D.-X., Reiter R.J., Chan Z., He C. (2015). Melatonin induces nitric oxide and the potential mechanisms relate to innate immunity against bacterial pathogen infection in Arabidopsis. J. Pineal Res..

[82] Masson P.H., Takahashi T., Angelini R. (2017). Editorial: Molecular mechanisms underlying polyamine functions in plants. Front. Plant Sci..

[83] Gill S.S., Tuteja N. (2010). Polyamines and abiotic stress tolerance in plants. Plant Signal. Behav..

[84] Sánchez-Rodríguez E., Romero L., Ruiz J.M. (2016). Accumulation of free polyamines enhances the antioxidant response in fruits of grafted tomato plants under water stress. J. Plant Physiol..

[85] Gong X., Shi S., Dou F., Song Y., Ma F. (2017). Exogenous melatonin alleviates alkaline stress in *Malus hupehensis* Rehd. by regulating the biosynthesis of polyamines. Molecules.

[86] Zhao H., Zhang K., Zhou X., Xi L., Wang Y., Xu H., Pan T., Zou Z. (2017). Melatonin alleviates chilling stress in cucumber seedlings by upregulation of CsZat12 and modulation of polyamine and abscisic acid metabolism. Sci. Rep..

[87] Back K., Tan D.-X., Reiter R.J. (2016). Melatonin biosynthesis in plants: Multiple pathways catalyze tryptophan to melatonin in the cytoplasm or chloroplasts. J. Pineal Res..

[88] Murch S.J., KrishnaRaj S., Saxena P.K. (2000). Tryptophan is a precursor for melatonin and serotonin biosynthesis in in vitro regenerated St. John’s wort (*Hypericum perforatum* L. cv. Anthos) plants. Plant Cell Rep..

[89] Byeon Y., Park S., Lee H.Y., Kim Y.-S., Back K. (2014). Elevated production of melatonin in transgenic rice seeds expressing rice tryptophan decarboxylase. J. Pineal Res..

[90] Zhao D., Wang R., Liu D., Wu Y., Sun J., Tao J. (2018). Melatonin and expression of tryptophan decarboxylase gene (TDC) in Herbaceous peony (*Paeonia lactiflora* Pall.) flowers. Molecules.

[91] Park S., Byeon Y., Back K. (2013). Transcriptional suppression of tryptamine 5-hydroxylase, a terminal serotonin biosynthetic gene, induces melatonin biosynthesis in rice (*Oryza sativa* L.). J. Pineal Res..

[92] Byeon Y., Choi G.-H., Lee H.Y., Back K. (2015). Melatonin biosynthesis requires *N*-acetylserotonin methyltransferase activity of caffeic acid *O*-methyltransferase in rice. J. Exp. Bot..

[93] Hardeland R. (2017). Taxon- and site-specific melatonin catabolism. Molecules.

[94] Kanwar M.K., Yu J., Zhou J. (2018). Phytomelatonin: Recent advances and future prospects. J. Pineal Res..

[95] Wei Y., Zeng H., Hu W., Chen L., He C., Shi H. (2016). Comparative transcriptional profiling of melatonin synthesis and catabolic genes indicates the possible role of melatonin in developmental and stress responses in rice. Front. Plant Sci..

[96] Byeon Y., Back K. (2015). Molecular cloning of melatonin 2-hydroxylase responsible for 2-hydroxymelatonin production in rice (Oryza sativa). J. Pineal Res..

[97] Zhang N., Zhang H.J., Zhao B., Sun Q.Q., Cao Y.Y., Li R., Wu X.X., Weeda S., Li L., Ren S. (2014). The RNA-seq approach to discriminate gene expression profiles in response to melatonin on cucumber lateral root formation. J. Pineal Res..

[98] Pang X., Wei Y., Cheng Y., Pan L., Ye Q., Wang R., Ruan M., Zhou G., Yao Z., Li Z. (2018). The Tryptophan Decarboxylase in *Solanum lycopersicum*. Molecules.

[99] Kang S., Kang K., Lee K., Back K. (2007). Characterization of rice tryptophan decarboxylases and their direct involvement in serotonin biosynthesis in transgenic rice. Planta.

[100] Fan J.B., Xie Y., Zhang Z.C., Chen L. (2018). Melatonin: A Multifunctional Factor in Plants. Int. J. Mol. Sci..

[101] Liu W., Zhao D., Zheng C., Chen C., Peng X., Cheng Y., Wan H. (2017). Genomic analysis of the ASMT gene family in *Solanum lycopersicum*. Molecules.

[102] Kang K., Lee K., Park S., Byeon Y., Back K. (2013). Molecular cloning of rice serotonin N-acetyltransferase, the penultimate gene in plant melatonin biosynthesis. J. Pineal Res..

[103] Byeon Y., Lee H.Y., Lee K., Park S., Back K. (2014). Cellular localization and kinetics of the rice melatonin biosynthetic enzymes SNAT and ASMT. J. Pineal Res..

[104] Kang S., Kang K., Lee K., Back K. (2007). Characterization of tryptamine 5-hydroxylase and serotonin synthesis in rice plants. Plant Cell Rep..

[105] Torrens-Spence M.P., Liu P., Ding H., Harich K., Gillaspy G., Li J. (2013). Biochemical evaluation of the decarboxylation and decarboxylation-deamination activities of plant aromatic amino acid decarboxylases. J. Biol. Chem..

[106] Torrens-Spence M.P., Lazear M., von Guggenberg R., Ding H., Li J. (2014). Investigation of a substrate-specifying residue within Papaver somniferum and Catharanthus roseus aromatic amino acid decarboxylases. Phytochemistry.

[107] Kang K., Kong K., Park S., Natsagdorj U., Kim Y.S., Back K. (2011). Molecular cloning of a plant N-acetylserotonin methyltransferase and its expression characteristics in rice. J. Pineal Res..

[108] Byeon Y., Lee H.Y., Back K. (2016). Cloning and characterization of the serotonin N-acetyltransferase-2 gene (SNAT2) in rice (*Oryza sativa*). J. Pineal Res..

[109] Maag J.L.V. (2018). gganatogram: An R package for modular visualisation of anatograms and tissues based on ggplot2. F1000Research.

[110] Ebisawa T., Karne S., Lerner M.R., Reppert S.M. (1994). Expression cloning of a high-affinity melatonin receptor from Xenopus dermal melanophores. Proc. Natl. Acad. Sci. USA.

[111] Ng K.Y., Leong M.K., Liang H., Paxinos G. (2017). Melatonin receptors: distribution in mammalian brain and their respective putative functions. Brain Struct. Funct..

[112] Witt-Enderby P.A., Bennett J., Jarzynka M.J., Firestine S., Melan M.A. (2003). Melatonin receptors and their regulation: biochemical and structural mechanisms. Life Sci..

[113] Dubocovich M.L., Delagrange P., Krause D.N., Sugden D., Cardinali D.P., Olcese J. (2010). International union of basic and clinical pharmacology. LXXV. Nomenclature, classification, and pharmacology of G protein-coupled melatonin receptors. Pharmacol. Rev..

[114] Wei J., Li D.-X., Zhang J.-R., Shan C., Rengel Z., Song Z.-B., Chen Q. (2018). Phytomelatonin receptor PMTR1-mediated signaling regulates stomatal closure in Arabidopsis thaliana. J. Pineal Res..

[115] Nosjean O., Ferro M., Cogé F., Beauverger P., Henlin J.-M., Lefoulon F., Fauchère J.-L., Delagrange P., Canet E., Boutin J.A. (2000). Identification of the Melatonin-binding SiteMT 3 as the Quinone Reductase 2. J. Biol. Chem..

[116] Zhang N., Sun Q., Zhang H., Cao Y., Weeda S., Ren S., Guo Y.-D. (2015). Roles of melatonin in abiotic stress resistance in plants. J. Exp. Bot..

[117] Tan D.X., Manchester L.C., Liu X., Rosales-Corral S.A., Acuna-Castroviejo D., Reiter R.J. (2013). Mitochondria and chloroplasts as the original sites of melatonin synthesis: a hypothesis related to melatonin’s primary function and evolution in eukaryotes. J. Pineal Res..

[118] Park S.-Y., B Seo S., J Lee S., G Na J., Kim Y.J. (2001). Mutation in PMR1, a Ca^2+^-ATPase in Golgi, confers salt tolerance in Saccharomyces cerevisiae by inducing expression of PMR2, an Na^+^-ATPase in plasma membrane. J. Biol. Chem..

